# Shades of white: The *Petunia* long corolla tube clade evolutionary history

**DOI:** 10.1590/1415-4757-GMB-2023-0279

**Published:** 2024-02-12

**Authors:** Alice Backes, Caroline Turchetto, Geraldo Mäder, Ana Lúcia A. Segatto, Sandro L. Bonatto, Loreta B. Freitas

**Affiliations:** 1 Universidade Federal do Rio Grande do Sul, Departamento de Genética, Porto Alegre, RS, Brazil.; 2 Universidade Federal do Rio Grande do Sul, Departamento de Botânica, Porto Alegre, RS, Brazil.; 3 Universidade Federal de Santa Maria, Departamento de Bioquímica e Biologia Molecular, Santa Maria, RS, Brazil.; 4 Pontifícia Universidade Católica do Rio Grande do Sul, A Escola de Ciências da Saúde e da Vida, Porto Alegre, RS, Brazil.

**Keywords:** Solanaceae, genetic variability, speciation, evolutionary relationships

## Abstract

Delimiting species is challenging in recently diverged species, and adaptive radiation is fundamental to understanding the evolutionary processes because it requires multiple ecological opportunities associated with adaptation to biotic and abiotic environments. The young *Petunia* genus (Solanaceae) is an excellent opportunity to study speciation because of its association with pollinators and unique microenvironments. This study evaluated the phylogenetic relationships among a *Petunia* clade species with different floral syndromes that inhabit several environments. We based our work on multiple individuals per lineage and employed nuclear and plastid phylogenetic markers and nuclear microsatellites. The phylogenetic tree revealed two main groups regarding the elevation of the distribution range, whereas microsatellites showed high polymorphism-sharing splitting lineages into three clusters. Isolation by distance, migration followed by new environment colonization, and shifts in floral syndrome were the motors for lineage differentiation, including infraspecific structuring, which suggests the need for taxonomic revision in the genus.

## Introduction

Adaptive radiation plays a fundamental role in our understanding of the evolutionary process, and it is frequently accepted that adaptive radiation requires multiple ecological opportunities associated with adaptation to biotic and abiotic environments (Gillespie et al., 2020). Criteria such as common ancestry, phenotype-driver selector, and rapid speciation have been proposed to identify adaptive radiation (Schluter, 2009). However, some authors consider it challenging to prove for most studies (Gillespie *et al.*, 2020). Delimiting species is difficult in recently derived species because of the short time interval since speciation could not be enough to accumulate genetic differentiation (e.g., Knowles and Carstens, 2007).

The genus *Petunia* (Solanaceae) encompasses 17 wild species distributed in southern South America (Greppi et al., 2019) and one of the most important ornamental plants, *P. hybrida.* Divided into two main clades based on molecular phylogenetic analysis (Reck-Kortmann et al., 2014), the genus has 14 bee-pollinated species that share several morphological traits, especially the corolla tube length, which is short, and the bluish pollen. Three other species display long corolla tubes and yellow pollen and are more variable in attracting different pollinators (Stehmann et al., 2009; Fregonezi et al., 2013). The ornamental species *P. hybrida* is considered a perfect supermodel for genetic and physiological studies (Vandenbussche et al., 2016), and the wild species might be excellent models for understanding the evolutionary process for young groups. The clades diverged ca. 2.8 Mya (Särkinen et al., 2013), and species in the short corolla clade colonized highland grasslands, diversifying ca. 1.0 Mya (Lorenz-Lemke et al., 2010).

The topology of *Petunia* phylogenetic trees profoundly changes when different molecular markers are considered. When based only on plastid markers, species are preferentially grouped according to their distribution in highlands (elevation up to 500 m above the sea level - a.s.l.) or lowlands (below 500 m high), respectively (Ando et al., 2005; Lorenz-Lemke et al., 2010). When the relationships are recovered based on only nuclear markers or combining nuclear and plastid sequences, the clades’ composition is supported by the corolla tube length, with the terminals’ position varying among gene trees (Chen et al., 2007; Kriedt et al., 2014; Reck-Kortmann et al., 2014; Segatto et al., 2016). 

The species in the short corolla tube clade (ST) share several morphological and ecological traits, and often it is difficult to distinguish them based only on morphology (Longo et al., 2014). The extensive genetic polymorphism sharing and some variable traits have promoted changes in the taxonomic classification of this group over time (Segatto et al., 2017). In the long corolla tube group (LT), the species are identified based on the corolla color (Stehmann et al., 2009), and no doubt has been put on their identity.

The diversification in each clade has been attributed to different main drivers. For species in the ST, especially those occupying higher elevations (ca. 900 m a.s.l. or more), it has been proposed an allopatric speciation, strongly influenced by climate changes during the late Pleistocene (Lorenz-Lemke et al., 2010; Barros et al., 2015, 2020). Pleistocene effects were also implicated in the intraspecific diversification of some species (Backes et al., 2019; Souza et al., 2022; Soares et al., 2023). Additionally, for ST lowland species (elevation < 500 m), ecological factors and geomorphology were the most important features, even when the species are parapatric (Ramos-Fregonezi et al., 2015; Segatto et al., 2017). The LT species show morphological traits associated with distinct floral syndromes, and the interaction with different pollinators is described as the main driver for diversification (Fregonezi *et al.*, 2013). 

The LT clade encompasses the species *P. axillaris*, divided into three subspecies [*P. axillaris* subsp. *axillaris*; *P. axillaris* subsp. *parodii,* and *P. axillaris* subsp. *subandina* - (hereafter shortly *P. axillaris*, *P. parodii*, and *P. subandina*, respectively)], *P. exserta*, *P. secreta*, and *P. occidentalis*. The *P. axillaris* subspecies display white flowers that are moth-pollinated (Ando et al., 1995; Venail et al., 2010); the bright red color and flower morphology of *P. exserta* attract hummingbirds (Stehmann et al., 2009); *P. secreta* shows pink corollas and is a bee-pollinated species (Rodrigues et al., 2018). The morphology of *P. occidentalis* corresponds to the melitophilous floral syndrome. However, no systematic pollination studies have been conducted with this taxon, and its effective pollinator is still unknown. 

Each taxon in LT shows different patterns of genetic structure throughout the geographic range (Segatto et al., 2014; Turchetto et al., 2014a,b, 2016; Giudicelli et al., 2022) and a complex process of intraspecific diversification emerges: *P. parodii* shows three main lineages, geographically structured (Chaco, Pampa-Brazil, and Pampa-Uruguay; Giudicelli *et al.*, 2022); *P. exserta* revealed two lineages with slight morphological variation and distribution (*P. exserta* E1 and *P. exserta* E2), each one occurring in a different rock formation in Serra do Sudeste; and *P. secreta* that would have two main genetic lineages (Turchetto *et al.*, 2016), more distinct from each other than canonical *P. secreta* is from *P. axillaris* (here treated as *P. secreta* and *P.* sp1, respectively)*.* An unnamed taxon (*P.* sp3) occurs close to *P. secreta* and *P. exserta* E1.

All taxa in LT have high levels of genetic polymorphism sharing (Kulcheski et al., 2006; Fregonezi et al., 2013; Reck-Kortmann et al., 2014; Turchetto et al., 2016), and interspecific hybridization has been observed among them (Lorenz-Lemke et al., 2006; Segatto et al., 2014; Turchetto *et al.*, 2015, 2019a, b; Giudicelli et al., 2019; Teixeira et al., 2019; Schnitzler et al., 2020; Caballero-Villalobos et al., 2021). Intraspecific morphological diversity was also observed (Turchetto *et al.*, 2016; Giudicelli *et al.*, 2019; Teixeira *et al.*, 2020), even in taxa that did not display differentiated genetic lineages as *P. axillaris* (Turchetto *et al.*, 2014b), which has a morphotype from coastal (A1) and another from inland (A2) distribution. 

Except for phylogenetic analyses, the LT taxa were not evaluated together based on their intra and interspecific genetic diversity. Thus, we aimed to (i) determine the phylogenetic relationships among taxa and intraspecific lineages in the long corolla tube clade of *Petunia* based on phylogenetic informative markers; (ii) compare the intraspecific genetic diversity among the LT taxa based on nuclear microsatellites; and (iii) identify any diversification process in course among LT lineages. We based our study on the cohesive species concept proposed by Templeton (1989) and as treated in Haselhorst et al. (2019).

## Material and Methods

### Phylogenetic approach

**Figure 1 -:**
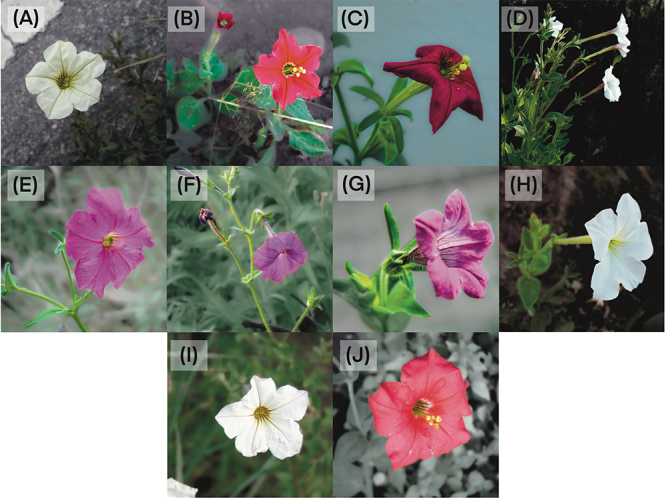
Representative individuals of each analyzed *Petunia* lineage. (A) *P. subandina*; (B) *P. exserta* E2; (C) *P.* sp3; (D) *P. axillaris* A2; (E) *P.* sp1; (F) *P. secreta*; (G) *P. occidentalis*; (H) *P. axillaris* A1; (I) *P. parodii*; (J) *P. exserta* E1.

We collected young and healthy leaves from multiple individuals of each LT lineage (Figure 1), except for *P. occidentalis,* for which we used an herbarium-derived sample (Table S1). We extracted the total DNA using the CTAB (cetyl-trimethyl ammonium bromide)-based method (Roy et al., 1992), evaluated DNA quality in a NanoDrop DN 1000 spectrophotometer (Thermo Fischer Scientific Co., Waltham, USA), and estimated the quantity using a Qubit fluorometer (Thermo Fischer). 

We amplified seven nuclear regions and five plastid DNA markers through PCR reactions using previously described primers and protocols (Table S2). We included once-obtained sequences (Reck-Kortmann et al., 2014) for some samples. We used two *Calibrachoa* species (Mäder and Freitas, 2019) and *P. integrifolia* representing the ST (Reck-Kortmann *et al.*, 2014) as outgroups. Amplicons were purified using a polyethylene glycol method (Dunn and Blattner, 1987) and sequenced in an ABI 3730XL (Thermo Fischer Sci.) sequencer.

We assembled and edited sequences using Chromas v.2.0 software (Technelysium, Helensvale, Australia) and prepared alignments per DNA marker using Muscle in MEGA X (Kumar et al., 2018) and concatenated them to the phylogenetic analyses. We manually edited the alignments when necessary and coded contiguous insertion/deletion (indels) events involving more than one base pair (bp) as one mutational event (Simmons and Ochoterena, 2000). We did not include ambiguous sites (more than one pick in the chromatogram) from nuclear markers in the final matrix (Mäder et al., 2010). One representative of each different sequence was deposited at GenBank (Table S3). We also used MEGA to estimate genetic diversity per marker (Table 1).

**Table 1 -:** Genetic diversity per marker used to obtain the phylogenetic tree for *Petunia* long corolla tube clade.

Genetic Marker	Alignment length	Variable sites (%)	PI sites (%)	Evolutionary Model*
*trnH-psbA* ^*1*^	424	20 (4.7)	9 (2.1)	HKY+G
*trnS-trnG* ^*1*^	652	15 (2.3)	9 (1.4)	HKY+G
*rps12-rpl20* ^*1*^	777	26 (3.4)	13 (1.7)	HKY+G
*trnL-rpl32* ^*1*^	976	25 (2.6)	13 (1.3)	HKY+G
*matK*	857	21 (2.5)	13 (1.5)	GTR+I
cpDNA Total	3,686	107 (2.9)	57 (1.6)	-
ITS	536	55 (10.3)	29 (5.4)	GTR+G
*Hf1*	1,395	50 (3.6)	24 (1.7)	GTR+G
*PolA1*	987	25 (2.5)	9 (0.9)	HKY+G
*G3pdh*	544	28 (5.2)	15 (2.8)	HKY+G
*PID3C4*	209	10 (4.8)	6 (2.9)	HKY+G
*WOX4*	231	43 (18.6)	32 (13.9)	HKY+G
*WUS*	206	31 (15.1)	18 (8.7)	HKY
nuDNA Total	4,108	242 (5.9)	133 (3.2)	-
**Total**	**7,794**	**349 (4.5)**	**190 (2.4)**	

PI - parsimoniously informative sites; *Best substitution model estimated with jModelTest based on Akaike information criterion; ^1^concatenated sequences

To estimate the evolutionary relationships among taxa and lineages, we used a Bayesian inference (BI) as implemented in BEAST v.1.10 (Suchard et al., 2018), assessing the tree support with posterior probability (PP) with 10^7^ chains. We estimated the best substitution model and gamma rate heterogeneity using jModelTest v.3.06 (Darriba et al., 2012) based on the Akaike information criterion (AIC) for each nuclear marker, *matK* gene, and combined intergenic plastid spacers, respectively (Table 1). We conducted BI analysis under the Yule process and two independent runs of 10 million generations, sampling every 1000 generations. We assessed Markov chain Monte Carlo (MCMC) convergence by examining effective sample size values (ESS > 200) and likelihood plots in Tracer v.1.7 (Rambaut et al., 2018). We discarded the initial 25% of trees as burn-in and summarized the remaining trees to generate a maximum clade credibility tree using TreeAnnotator v.1.7.5 (Suchard *et al.*, 2018) visualized with FigTree v.1.4.1 (http://tree.bio.ed.ac.uk/software/figtree/). PP ≥ 0.90 values were considered to represent strong support.

### Intraspecific variability

To estimate the intraspecific diversity, we amplified seven nuclear microsatellite loci (Table S4) for all taxa (except *P. occidentalis*), including individuals throughout the entire geographic distribution of each lineage, proportional to population density. We genotyped 10 *P. axillaris* A1, 63 *P. axillaris* A2, 13 *P. exserta* E1, 82 *P. exserta* E2, 50 *P. secreta*, 39 *P. parodii*, 23 *P. subandina*, 23 *P.* sp1, and 11 *P.* sp3. We visualized and scored the alleles with GeneMarker v.1.97 software (Softgenetics LLC, State College, USA) and used Micro-Checker (van Oosterhout et al., 2004) software to identify possible null alleles, significant allele dropout, and scoring errors due to stutter peaks.

We used the FSTAT v.2.9.3.2 software (Goudet, 1995) to evaluate the number of alleles per locus (A) and Nei’s unbiased gene diversity (GD; Nei, 1987). Additionally, we used AZDE (Szpiech et al., 2008) to estimate allelic richness (AR) and number of private alleles (PA) through rarefaction, as sample sizes vary among lineages.

We conducted a discriminant analysis of principal components (DAPC; Jombart et al., 2010) employed in the R program for Statistical Computing v.3.3.2 (R Core Team, 2020) to explore genetic groups. The lowest Bayesian information criterion (BIC) in DAPC was used to assess the best number of groups, and we did not include taxonomic and geographic prior information.

## Results

### Evolutionary relationships

**Figure 2 -:**
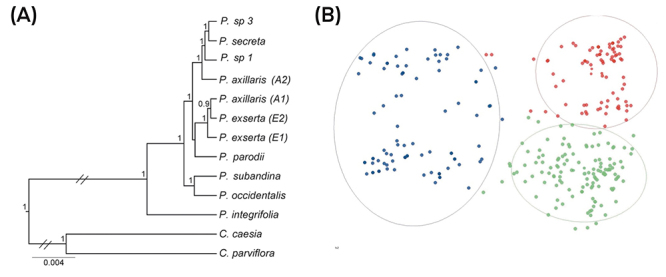
Evolutionary relationships among *Petunia* long corolla tube clade. (A) Bayesian inference phylogenetic tree including plastid and nuclear sequences. Each branch represents collapsed individuals with identical sequences. (B) Cartesian plane obtained in DAPC analysis based on nuclear microsatellites (best K = 3). Colors indicate clusters: red, cluster 1; green, cluster 2; and blue, cluster 3. Cluster composition in lineages and individual numbers follow Table S5.

We obtained a data matrix with 7,794 characters based on the DNA markers, from which ~5% were variable, and ~3% were parsimoniously informative. Nuclear regions were more variable and informative than plastid markers (Table 1). The BI analysis (Figure 2A) split the species with long corolla tubes in two main clades, mainly based on elevation: clade I, species distributed in elevations higher than 700 m a.s.l (*P. subandina* and *P. occidentalis*), and clade II, species found at less than 700 m a.s.l (remain lineages). Clade II also could be divided into two subclades, IIA encompassing *P. secreta, P.* sp1, *P.* sp3, and the inland lineage of *P. axillaris*. In subclade IIB, we found coastal *P. axillaris* lineage, two *P. exserta* lineages, and *P. parodii.* These ten lineages were well supported (PP ≥ 0.90), except for the *P. parodii* positioning (PP < 0.90). The separation between *Petunia* LT and ST clades was confirmed.

### Intraspecific variability

Considering the seven SSR loci, all individuals exhibited a maximum of two alleles per locus, as expected for diploid species, and the sizes of the alleles were compatible with the repetition for each locus. All loci were polymorphic among lineages. The most variable lineage was *P. axillaris* A1, considering AR and GD indices, whereas the least variable was *P.* sp1. The highest number of private alleles (PA) was observed in *P. subandina*, whereas *P. exserta* E1 has the lowest (Table 2). 

**Table 2 -:** Median values for genetic diversity indices observed in *Petunia* long corolla tube lineages based on seven nuclear microsatellites

Lineages	N	A	AR	PA	GD
*P. axillaris* A1	10	6.3	6.3	0.68	0.74
*P. axillaris* A2	63	8.1	5.5	0.25	0.70
*P. exserta* E1	13	3.7	3.5	0.06	0.54
*P. exserta* E2	82	6.7	4.5	0.59	0.57
*P. secreta*	50	7.6	5.2	0.43	0.62
*P. parodii*	39	6.3	4.7	0.37	0.64
*P. subandina*	23	4.9	4.3	0.70	0.69
*P.* sp1	23	4.1	3.2	0.38	0.43
*P.* sp3	11	4.7	4.7	0.11	0.67

N - number of analyzed individuals; A - total number of alleles per species; AR - allele richness; PA - number of private alleles; GD - gene diversity.

The DAPC analysis (Figure 2B), including all individuals and microsatellite loci, revealed the most probable K = 3 groups. Individuals of most lineages were distributed in two or three groups, except *P.* sp1, from which all individuals belonged to the first cluster. Approximately 50% of *P. subandina, P. parodii*, and *P. axillaris* A2 samples composed the first cluster. The second cluster encompassed most *P. exserta* E1 and E2, and *P.* sp3 individuals, whereas all lineages had representatives in group 3, except *P.* sp1. Most *P. secreta* and *P. axillaris* A1 belonged to the third group (Table S5). The polymorphism sharing based on microsatellite alleles did not replicate the evolutionary relationships among species. Groups were homogeneous with low superimposition in the Cartesian plane.

## Discussion

Here, we investigated the evolutionary relationships among the *Petunia* long corolla tube species employing a phylogenetic approach and intraspecific genetic variability. The taxa in the LT clade display marked differentiation in floral traits associated with pollinator attraction (Stehmann et al., 2009), and plant-pollinator interaction was proposed as the main speciation driver in the group (Fregonezi et al., 2013). Despite attracting different pollinators, several hybrid populations are found (e.g., Turchetto et al., 2019b; Giudicelli et al., 2019).

Our results revealed unexpected relationships regarding previous studies (e.g., Reck-Kortmann et al., 2014). On the other hand, the present work is the first to include multiple samples and intraspecific lineages throughout their entire geographic distribution. In the *Petunia* genus, geographic isolation is often implicated in population structure and reproductive isolation (e.g., Giudicelli et al., 2022; Guzmán et al., 2022). Moreover, local adaptation and microenvironmental conditions keep species limits (e.g., Segatto et al., 2017; Caballero-Villalobos et al., 2021), contributing to differentiation (Fregonezi et al., 2013; Pezzi et al., 2022).

The phylogenetic tree and SSR-based analyses were not entirely congruent. Phylogenetic markers indicated with full support the split between high elevation-distributed species (*P. occidentalis* and *P. subandina*) and the lowland species (remaining lineages, all distributed at < 500 m a.s.l.), whereas SSR profiles formed three groups that did not reflect phylogenetic clades and subclades. SSR-based group 2 encompassed all *P. exserta* individuals, independently of their occurrence area, most *P.* sp3, one *P. secreta* from the same region than *P.* sp3, and one *P. axillaris* A2 sampled close to *P. exserta. Petunia exserta* occupies the subclade IIB in the tree, whereas the remaining lineages from group 2 form the subclade IIA. In turn, groups 1 and 3 clustered individuals of all lineages in different proportions (except for *P.* sp1, which integrates only group 1): *P. axillaris* A2, *P. parodii*, and *P. subandina* were equally distributed between groups 1 and 3, whereas *P. axillaris* A1 and *P. secreta* mainly integrated the group 3. The lineages *P. axillaris* A1 and *P. secreta* were not closely related in the phylogenetic tree, occupying different subclades despite the high similarity in their SSR profiles. The geographic distribution of *P. axillaris* A1 is on the southern Atlantic coast, predominantly in Uruguay (Turchetto et al., 2014a), whereas *P. secreta* is endemic to Serra do Sudeste in Rio Grande do Sul (Stehmann and Semir, 2005).

Almost all phylogenetic analyses including the LT taxa (Ando et al., 2005; Kriedt et al., 2014; Reck-Kortmann et al., 2014; Segatto et al., 2016) placed *P. subandina* and *P. occidentalis* as sister species (but also see Chen et al., 2007), despite the first displays long corolla tube and yellow pollen as the remaining species in the LT, whereas *P. occidentalis* shows a short corolla tube and bluish pollen as all species in the ST. Regarding the geographical distribution, *P. occidentalis* is restricted to the sub-Andean region, in elevation up to 900 m, and isolated from the other *Petunia* species by the Chaco (Tsukamoto et al., 1998); the remaining species in LT are found in grasslands in Chaco or Pampa (Stehmann et al., 2009), in open rocky ground areas and roadside slopes, except for *P. subandina,* which occurs only in the sub-Andean mountains (Ando, 1996). The taxa *P. axillaris*, *P. exserta*, and *P. secreta* occur in sympatry in Brazil. However, *P. axillaris* is widely distributed in the Uruguayan Pampa, whereas the other two species are narrowly endemic to rocky formations in southern Brazil. The *P. parodii* can be found in Chaco (Argentina) and Pampa (southern Brazil, Uruguay, and Argentina), where the plants grow disjunct from *P. axillaris*. Except for *P. subandina* and *P. occidentalis*, the species in LT are distributed from zero to less than 500 m a.s.l., occupying areas proposed as ancestral for the *Petunia* genus (Reck-Kortmann *et al.*, 2014).

The most surprising result was the divergence between *P. axillaris* interspecific lineages A1 and A2. According to the phylogenetic markers, this taxon was paraphyletic. Previous works (Turchetto et al., 2014a, b) support the separation found here among *P. axillaris*, *P. parodii,* and *P. subandina,* indicating they should be treated as independent evolutionary units and not only as subspecies. Although *P. axillaris*, *P. parodii,* and *P. subandina* shared several plastid haplotypes and no genetic-based intraspecific groups have been found (Turchetto *et al.*, 2014a), morphologic floral traits revealed that *P. axillaris* can be divided in two groups that correspond to coastal (A1 in the present work) and inland (A2) populations. In the same way, ecological features pointed to the same *P. axillaris* subgroups and three groups in *P. parodii* (Chaco, Pampa-Brazil, and Pampa-Uruguay), which were not perceived based on morphologic analysis. The *P. parodii* subdivision was not confirmed here in the phylogenetic tree and SSR, but it was also identified using a sizeable genomic evaluation (Giudicelli et al., 2022). 

It is widely accepted that ecological divergence due to habitat differences plays an essential role in lineage differentiation (e.g., Foster et al., 2007), notably regarding to adaptation to extreme environments such as coastal areas (e.g., Lowry et al., 2008) that are often reflected in morphological traits in addition to genetic markers. Significant morphologic differences were already observed comparing *P. axillaris* inland populations in Brazil with coastal populations from Uruguay, whereas *P. parodii* Brazilian populations were not different from those collected in Uruguay (Kokubun et al., 2006). Such differences or their absence followed taxa’s self(in)-compatibility system.

The polymorphism sharing between some lineages in the *Petunia* LT can be explained by introgression due to hybrid populations’ high frequency and stability (e.g., Schnitzler et al., 2020), whereas others are based on shared ancestry. Hybridization could be discarded because of the long distance between populations, such as *P. exserta* and *P. axillaris* A1 or *P. subandina* and all others, as the distance between populations exceeds 1 km, which is the maximum estimated distance for pollen dispersal (e.g., Turchetto et al., 2015, 2022; Rodrigues et al., 2019). Moreover, seed dispersal in *Petunia* is very limited, with seeds falling close to the mother plant by autochory (Stehmann et al., 2009).

**Figure 3 -:**
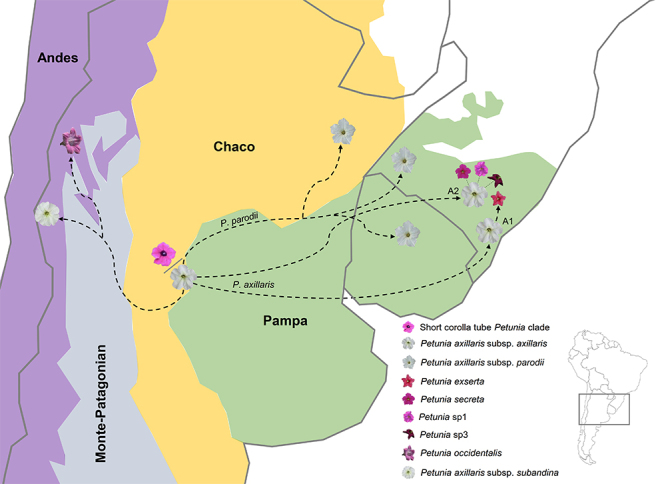
Putative migratory routes and diversification for *Petunia* corolla tube clade species.

The evolutionary relationships and polymorphism-sharing in the *Petunia* long corolla tube clade could be explained based on the migration routes (Figure 3) from an albino ancestor (Wijsman, 1983), which originated in lowland (Reck-Kortmann et al., 2014), ca. 2.8 Mya (Särkinen et al., 2013), with subsequent diversification after colonized new environments or under pollinator selection (Fregonezi et al., 2013). The albino lineage arose from the anthocyanin 2 (*AN2*) gene inactivation. The *AN2* is active in the species of the ST clade and responsible for the pink color (Quattrocchio et al., 1999), the critical morphologic trait to attract bees. The ST species probably represent the genus ancestor, which appeared in lowlands in southern South America, likely in the Pampa (Reck-Kortmann *et al.*, 2014). The genus diverged from the sister group ca. 8.0 Mya (Särkinen *et al.*, 2013).

The first step in LT clade differentiation was the highlands’ colonization, which also explains the presence of *P. occidentalis* in the clade despite its several morphological traits in common with ST species. *Petunia occidentalis* could represent an incomplete lineage sorting in the highland LT clade, sister of the albino *P. subandina*. The albino lineage would expand its distribution towards the southern South American grasslands as the Pleistocene climate changes allowed. The albino lineage colonized the Chaco, migrating to the north, and Pampa, growing to the south and east, nowadays represented by *P. parodii* (Giudicelli et al., 2022) and its parapatric lineage *P. axillaris* A2. 

These last two lineages, *P. parodii* and *P. axillaris* A2, could have given rise to the colored lineages in the clade as they advanced colonizing new environments. The albino *P. parodii* and *P. axillaris* A1 and the red-flowered *P. exserta* share several polymorphisms (e.g., Segatto et al., 2014; Li et al., 2023), despite currently not being found close. Mainly regarding *P. exserta*, this species inhabits a very particular microenvironment, inside small caves where plants grow protected from direct sunlight and rain (Stehmann et al., 2009; Segatto *et al.*, 2014), an inhospitable environment for other *Petunia* species. The two *P. exserta* lineages (E1 and E2) differ mainly in flower color hue (Figure 1) and distribution as each inhabits a different rock formation. *Petunia exserta* E2 is sympatric to some *P. axillaris* A2 populations, whereas *P. exserta* E1 occurs in the same formation as *P. secreta*. The red color of *P. exserta* petals is reached through a complex gene interaction that begins with a moderate upregulation and shifts in tissue specificity of the *Deep Purple* gene that restores anthocyanin biosynthesis (Berardi et al., 2021). *P. exserta* retains the same nonfunctional *AN2* copy present in *P. axillaris.*

The pink-flowered *P. secreta* and *P.* sp1 differ from *P. axillaris* only based on the flower color (Stehmann and Semir, 2005), and this difference is due to the regain in *AN2* gene function (Esfeld et al., 2018). *Petunia secreta* and *P*. sp1 occur in the same geographic area as *P. axillaris* A2. Still, whereas *P.* sp1 occupies a similar environment closely distributed to *P. axillaris*, *P. secreta* is found ca. 40 Km away from the closest *P. axillaris* A2 population and in an entirely diverse microenvironment (Turchetto et al., 2016; Rodrigues et al., 2019). *Petunia* sp3 is the *P. secreta* sister lineage, despite being morphologically similar to *P. exserta,* mainly regarding the exserted styles and anthers (Figure 1). Indeed, *P.* sp3, *P. secreta*, and *P. exserta* E1 are endemic to the same rock formation. Still, whereas *P. exserta* E1 occupies shaded locations, *P. secreta* and *P.* sp3 individuals grow in sunny places. Our results did not discard a hybrid status for *P.* sp3.

In conclusion, we described the evolutionary relationships among the *Petunia* long corolla tube clade due to ancestral geographic expansion with local adaptation and pollinator interaction as the vital diversification drivers. Structuring in LT lineages depends on isolation by distance, and high polymorphism-sharing is due to a common ancestor and rapid adaptive radiation.

## Supplementary material

The following online material is available for this article:

Table S1 - Sampling information for *Petunia* long corolla tube clade and outgroups.

Table S2 - Genetic markers that were used to obtain the phylogenetic tree for the *Petunia* long corolla tube clade.

Table S3 - GenBank numbers for phylogenetic markers.

Table S4 - Nuclear microsatellite markers used to genotype *Petunia* long corolla tube clade.

Table S5 - Number of individuals per lineage per DAPC group (best K = 3).

## References

[B1] Ando T (1996). Distribution of Petunia axillaris (Solanaceae) and its new subspecies in Argentina and Bolivia. Acta Fitotaxon Geobot.

[B2] Ando T, Lida S, Kokubun H, Ueda Y, Marchesi E (1995). Distribution of Petunia axillaris sensu lato in Uruguay as revealed by discriminant analysis of the live plants. J Japan Soc Hort Sci.

[B3] Ando T, Kokubun H, Watanabe H, Tanaka N, Yukawa T, Hashimoto G, Marchesi E, Suárez E, Basualdo IL (2005). Phylogenetic analysis of Petunia sensu Jussieu (Solanaceae) using chloroplast DNA RFLP. Ann Bot.

[B4] Backes A, Mäder G, Turchetto C, Segatto ALA, Fregonezi JN, Bonatto SL, Freitas LB (2019). How diverse can rare species be on the margins of genera distribution?. AoB Plants.

[B5] Barros MJF, Silva-Arias GA, Fregonezi JN, Turchetto-Zolet AC, Iganci JRV, Diniz-Filho JAF, Freitas LB (2015). Environmental drivers of diversity in subtropical highland grasslands: A comparative analysis of Adesmia, Calibrachoa, and Petunia. Perspect Plant Ecol Evol Syst.

[B6] Barros MJF, Silva-Arias GA, Segatto ALA, Reck-Kortmann M, Fregonezi JN, Diniz-Filho JAF, Freitas LB (2020). Phylogenetic niche conservatism and plant diversification in South American subtropical grasslands along multiple climatic dimensions. Genet Mol Biol.

[B7] Berardi AE, Esfeld K, Jäggi L, Mandel T, Cannarozzi GM, Kuhlemeier C (2021). Complex evolution of novel red floral color in Petunia. Plant Cell.

[B8] Caballero-Villalobos L, Silva-Arias GA, Turchetto C, Giudicelli GC, Petzold E, Bombarely A, Freitas LB (2021). Neutral and adaptive genomic variation in hybrid zones of two ecologically diverged Petunia species (Solanaceae). Bot J Lin Soc.

[B9] Chen S, Matsubara K, Omori T, Kokubun H, Kodama H, Watanabe H, Hashimoto G, Marchesi E, Bullrich L, Ando T (2007). Phylogenetic analysis of the genus Petunia (Solanaceae) based on the sequence of the Hf1 gene. J Plant Res.

[B10] Darriba D, Taboada GL, Doallo R, Posada D (2012). jModelTest 2: More models, new heuristics and parallel computing. Nat Methods.

[B11] Dunn IS, Blattner FR (1987). Charons 36-40: Multi-enzyme, high capacity, recombination deficient replacement vectors with polylinkers and polystuffers. Nucleic Acids Res.

[B12] Esfeld K, Berardi AE, Moser M, Bossolini E, Freitas L, Kuhlemeier C (2018). Pseudogenization and resurrection of a speciation gene. Curr Biol.

[B13] Foster SA, McKinnon GE, Steane DA, Potts BM, Vaillancourt RE (2007). Parallel evolution of dwarf ecotypes in the forest tree Eucalyptus globulus. New Phytol.

[B14] Fregonezi JN, Turchetto C, Bonatto SL, Freitas LB (2013). Biogeographical history and diversification of Petunia and Calibrachoa (Solanaceae) in the Neotropical Pampas grassland. Bot J Lin Soc.

[B15] Gillespie RG, Bennett GM, de Meester L, Feder JL, Fleischer RC, Harmon LJ, Hendry AP, Knops ML, Mallet J, Martin C (2020). Comparing adaptive radiations across space, time, and taxa. J Hered.

[B16] Giudicelli GC, Turchetto C, Teixeira MC, Freitas LB (2019). Morphological and genetic characterisation in putative hybrid zones of Petunia axillaris subsp. axillaris and subsp. parodii (Solanaceae). Bot J Lin Soc.

[B17] Giudicelli GC, Turchetto C, Guzmán-Rodriguez S, Teixeira MC, Petzold E, Bombarely A, Freitas LB (2022). Population genomics indicates micro-refuges and riverine barriers for a southern South American grassland nightshade. J Biogeogr.

[B18] Goudet J (1995). FSTAT version 1.2: A computer program to calculate F-statistics. J Hered.

[B19] Greppi JA, Hagiwara JC, Stehmann JR (2019). A new species of Petunia (Solanaceae) from Corrientes, Argentina. Phytotaxa.

[B20] Guzmán S, Giudicelli GC, Turchetto C, Bombarely A, Freitas LB (2022). Neutral and outlier single nucleotide polymorphisms disentangle the evolutionary history of a coastal Solanaceae species. Mol Ecol.

[B21] Haselhorst MSH, Parchman TL, Buerkle CA (2019). Genetic evidence for species cohesion, substructure and hybrids in spruce. Mol Ecol.

[B22] Jombart T, Devillard S, Balloux F (2010). Discriminant analysis of principal components: A new method for the analysis of genetically structured populations. BMC Genet.

[B23] Knowles LL, Carstens BC (2007). Delimiting species without monophyletic gene trees. Syst Biol.

[B24] Kokubun H, Nakano M, Tsukamoto T, Watanabe H, Hashimoto G, Marchesi E, Bullrich L, Basualdo IL, Kao T-H, Ando T (2006). Distribution of self-compatible and self-incompatible populations of Petunia axillaris (Solanaceae) outside Uruguay. J Plant Res.

[B25] Kriedt RA, Cruz GMQ, Bonatto SL, Freitas LB (2014). Novel transposable elements in Solanaceae: Evolutionary relationships among Tnt1-related sequences in wild Petunia species. Plant Mol Biol Rep.

[B26] Kulcheski FR, Muschner VC, Lorenz-Lemke AP, Stehmann JR, Bonatto SL, Salzano FM, Freitas LB (2006). Molecular phylogenetic analysis of Petunia Juss. (Solanaceae). Genetica.

[B27] Kumar S, Stecher G, Li M, Knyaz C, Tamura K (2018). MEGA X: Molecular evolutionary genetics analysis across computing platforms. Mol Biol Evol.

[B28] Li C, Binaghi M, Pichon V, Cannarozzi G, Freitas LB, Hanemian M, Kuhlemeier C (2023). Tight genetic linkage of genes causing hybrid necrosis and pollinator isolation between young species. Nat Plants.

[B29] Longo D, Lorenz-Lemke AP, Mäder G, Bonatto SL, Freitas LB (2014). Phylogeography of the Petunia integrifolia complex in southern Brazil. Bot J Lin Soc.

[B30] Lorenz-Lemke AP, Mäder G, Muschner VC, Stehmann JR, Bonatto SL, Salzano FM, Freitas LB (2006). Diversity and natural hybridization in a highly endemic species of Petunia (Solanaceae): A molecular and ecological analysis. Mol Ecol.

[B31] Lorenz-Lemke AP, Togni PD, Mäder G, Kriedt RA, Stehmann JR, Salzano FM, Bonatto SL, Freitas LB (2010). Diversification of plant species in a subtropical region of eastern South American highlands: A phylogeographic perspective on native Petunia (Solanaceae). Mol Ecol.

[B32] Lowry DB, Rockwood RC, Willis JH (2008). Ecological reproductive isolation of coast and inland races of Mimulus guttatus. Evolution.

[B33] Mäder G, Freitas LB (2019). Biogeographical, ecological, and phylogenetic analyses clarifying the evolutionary history of Calibrachoa in South American grasslands. Mol Phylogen Evol.

[B34] Mäder G, Zamberlan PM, Fagundes NJ, Magnus T, Salzano FM, Bonatto SL, Freitas LB (2010). The use and limits of ITS data in the analysis of intraspecific variation in Passiflora L. (Passifloraceae). Genet Mol Biol.

[B35] Nei M (1987). Molecular evolutionary genetics.

[B36] Pezzi PH, Guzmán-Rodriguez S, Giudicelli GC, Turchetto C, Bombarely A, Freitas LB (2022). A convoluted tale of hybridization between two Petunia species from a transitional zone in South America. Perspec Plant Ecol Evol Syst.

[B37] Quattrocchio F, Wing J, van der Woude K, Souer E, de Vetten N, Mol J, Koes R (1999). Molecular analysis of the anthocyanin2 gene of Petunia and its role in the evolution of flower color. Plant Cell.

[B38] R Core Team (2020). R: A language and environment for statistical computing.

[B39] Rambaut A, Drummond AJ, Xie D, Baele G, Suchard MA (2018). Posterior summarisation in Bayesian phylogenetics using Tracer 1.7. Syst Biol.

[B40] Ramos-Fregonezi AMC, Fregonezi JN, Cybis GB, Fagundes NJR, Bonatto SL, Freitas LB (2015). Were sea level changes during the Pleistocene in the South Atlantic coastal plain a driver of speciation in Petunia (Solanaceae)?. BMC Evol Biol.

[B41] Reck-Kortmann M, Silva-Arias GA, Segatto ALA, Mäder G, Bonatto SL, Freitas LB (2014). Multilocus phylogeny reconstruction: New insights into the evolutionary history of the genus Petunia. Mol Phylogenet Evol.

[B42] Rodrigues DM, Caballero-Villalobos L, Turchetto C, Jacques RA, Kuhlemeier C, Freitas LB (2018). Do we truly understand pollination syndromes in Petunia as much as we suppose?. AoB Plants.

[B43] Rodrigues DM, Turchetto C, Lima JS, Freitas LB (2019). Diverse yet endangered: Pollen dispersal and mating system reveal inbreeding in a narrow endemic plant. Plant Ecol Divers.

[B44] Roy A, Frascaria N, MacKay J, Bousquet J (1992). Segregating random ampliﬁed polymorphic DNAs (RAPDs) in Betula alleghaniensis. Theor Appl Genet.

[B45] Särkinen T, Bohs L, Olmstead RG, Knapp S (2013). A phylogenetic framework for evolutionary study of the nightshades (Solanaceae): A dated 1000-tip tree. BMC Evol Biol.

[B46] Schluter D (2009). Evidence for ecological speciation and its alternative. Science.

[B47] Schnitzler CK, Turchetto C, Teixeira MC, Freitas LB (2020). What could be the fate of secondary contact zones between closely related plant species?. Genet Mol Biol.

[B48] Segatto ALA, Cazé ALR, Turchetto C, Klahre U, Kuhlemeier C, Bonatto SL, Freitas LB (2014). Nuclear and plastid markers reveal the persistence of genetic identity: A new perspective on the evolutionary history of Petunia exserta. Mol Phylogenet Evol.

[B49] Segatto ALA, Thompson CE, Freitas LB (2016). Contribution of WUSCHEL-related homeobox (WOX) genes to identify the phylogenetic relationships among Petunia species. Genet Mol Biol.

[B50] Segatto ALA, Reck-Kortmann M, Turchetto C, Freitas LB (2017). Multiple markers, niche modelling, and bioregions analyses to evaluate the genetic diversity of a plant species complex. BMC Evol Biol.

[B51] Simmons MP, Ochoterena H (2000). Gaps as characters in sequence-based phylogenetic analyses. Syst Biol.

[B52] Soares LS, Fagundes NJR, Freitas LB (2023). Past climate changes and geographical barriers: The evolutionary history of a subtropical highland grassland Solanaceae species. Bot J Lin Soc.

[B53] Souza AC, Giudicelli GC, Teixeira MC, Turchetto C, Bonatto SL, Freitas LB (2022). Genetic diversity in micro-endemic plants from highland grasslands in southern Brazil. Bot J Lin Soc.

[B54] Stehmann JR, Semir J (2005). New species of Calibrachoa and Petunia (Solanaceae) from subtropical South America. Monogr Syst.

[B55] Stehmann JR, Lorenz-Lemke AP, Freitas LB, Semir J, Gerats T, Strommer J (2009). *Petunia* evolutionary, developmental and physiological genetics.

[B56] Suchard MA, Lemey P, Baele G, Ayres DL, Drummond AJ, Rambaut A (2018). Bayesian phylogenetic and phylodynamic data integration using BEAST 1.10. Virus Evol.

[B57] Szpiech ZA, Jakobsson M, Rosenberg NA (2008). ADZE: A rarefaction approach for counting alleles private to combinations of populations. Bioinformatics.

[B58] Teixeira MC, Turchetto C, Hartke S, Schnitzler CK, Freitas LB (2019). Morphological and genetic perspectives of hybridisation in two contact zones of closely related species of Petunia (Solanaceae) in southern Brazil. Acta Bot Bras.

[B59] Teixeira MC, Turchetto C, Maestri R, Freitas LB (2020). Morphological characterisation of sympatric and allopatric Petunia exserta and Petunia axillaris (Solanaceae) populations. Bot J Lin Soc.

[B60] Templeton AR, Otte D, Endler JA (1989). Speciation and its consequences.

[B61] Tsukamoto T, Ando T, Kokubun H, Watanabe H, Tanaka R, Hashimoto G, Marchesi E, Kao T (1998). Differentiation in the status of self-incompatibility among all natural taxa of Petunia (Solanaceae). Acta Phytotax Geobot.

[B62] Turchetto C, Fagundes NJR, Segatto ALA, Kuhlemeier C, Solís-Neffa VG, Speranza PR, Bonatto SL, Freitas LB (2014). Diversification in the South American Pampas: The genetic and morphological variation of the widespread Petunia axillaris complex (Solanaceae). Mol Ecol.

[B63] Turchetto C, Segatto ALA, Telles MPC, Diniz-Filho JAF, Freitas LB (2014). Infraspecific classification reflects genetic differentiation in the widespread Petunia axillaris complex: a comparison among morphological, ecological, and genetic patterns of geographic variation. Perspect Plant Ecol Evol Syst.

[B64] Turchetto C, Segatto ALA, Beduschi J, Bonatto SL, Freitas LB (2015). Genetic differentiation and hybrid identification using microsatellite markers in closely related wild species. AoB Plants.

[B65] Turchetto C, Segatto ALA, Mäder G, Rodrigues DM, Bonatto SL, Freitas LB (2016). High levels of genetic diversity and population structure in an endemic and rare species: Implications for conservation. AoB Plants.

[B66] Turchetto C, Schnitzler CK, Freitas LB (2019). Species boundary and extensive hybridization and introgression in Petunia. Acta Bot Bras.

[B67] Turchetto C, Segatto ALA, Silva-Arias GA, Beduschi J, Kuhlemeier C, Bonatto SL, Freitas LB (2019). Contact zones and their consequences: Hybridization between two ecologically isolated wild Petunia species. Bot J Lin Soc.

[B68] Turchetto C, Segatto ALA, Turchetto-Zolet AC (2022). Biotic and abiotic factors in promoting the starting point of hybridization in the Neotropical flora: Implications for conservation in a changing world. Bot J Lin Soc.

[B69] van Oosterhout C, Hutchinson WF, Willis DPM, Shipley P (2004). Micro-checker: Software for identification and correcting genotyping errors in microsatellite data. Mol Ecol Notes.

[B70] Vandenbussche M, Chambrier P, Bento SR, Morel P (2016). Petunia, your next supermodel?. Front Plant Sci.

[B71] Venail J, Dell’Olivo A, Kuhlemeier C (2010). Speciation genes in the genus Petunia. Philos Trans Roy Soc B.

[B72] Wijsman HJW (1983). On the interrelationships of certain species of Petunia. II. Experimental data: Crosses between different taxa. Acta Bot Neerl.

