# Supplementary material for: Shades of white: The Petunia long corolla tube clade evolutionary history
Source: Genet Mol Biol. 2024 Feb 12;47(1):e20230279. doi: 10.1590/1415-4757-GMB-2023-0279 (PMC10882218; doi:10.1590/1415-4757-GMB-2023-0279)
Supplement: Table S1 - [file 1415-4757-GMB-47-01-e20230279-s1.pdf]

Genetics and Molecular Biology
https://doi.org/10.1590/1678-4685-GMB-2023-0279

Supplementary Material to "Shades of white: the Petunia long corolla tube clade evolutionary history"

Table S1 - Sampling information for *Petunia* long corolla tube clade and outgroups.

Lineage code	Geographic coordinates
P. axillaris (A1)	34° 54' 48"S, 55° 02' 545"W
P. axillaris (A2)	30° 12' 55"S, 52° 33' 55"W
P. parodii	30º 00' 55" S, 56º 26' 02" W
P. subandina	31° 45' 02"S, 64° 55' 51"W
P. exserta (E1)	30° 32' 43"S, 53° 33' 09"W
P. exserta (E2)	30° 50' 09"S, 53° 30' 24"W
P. secreta	30° 32' 47"S, 53° 33' 04"W
P. sp1	30° 21' 19"S, 53° 28' 43"W
P. sp3	30° 32' 48"S, 53° 33' 06"W
P. occidentalis	24° 11' 40"S, 65° 17' 49"W
P. integrifolia	30° 53' 27"S, 53° 31' 58"W
C. caesia	26° 12' 27"S, 48° 18' 31"W
C. parviflora	30° 04' 13"S, 51° 07' 15"W
