# Supplementary material for: Shades of white: The Petunia long corolla tube clade evolutionary history
Source: Genet Mol Biol. 2024 Feb 12;47(1):e20230279. doi: 10.1590/1415-4757-GMB-2023-0279 (PMC10882218; doi:10.1590/1415-4757-GMB-2023-0279)
Supplement: Table S2 - [file 1415-4757-GMB-47-01-e20230279-s2.pdf]

Genetics and Molecular Biology
https://doi.org/10.1590/1678-4685-GMB-2023-0279

Supplementary Material to "Shades of white: the Petunia long corolla tube clade evolutionary history"

Table S2 - Genetic markers used to obtain the phylogenetic tree for *Petunia* long corolla tube clade

Genome	Genetic marker	Primers and protocols reference
cpDNA	*trnH-psbA*[#]	Sang *et al.* (1997)
	trnS-trnG[#]	Hamilton (1999)
	rps12-rpl20[#]	Shaw *et al.* (2005)
	trnL-rpl32[#]	Shaw *et al.* (2007)
	matK gene	Johnson and Soltis (1994)
Nuclear	ITS	Desfeux and Lejeune (1996)
	Hf1b gene	Reck-Kortmann *et al.* (2014)
	PolA1 gene	Zhang *et al.* (2008)
	G3pdh	Olsen and Schaal (1999)
	PID3C4	Kriedt *et al.* (2011)
	WOX4 intron	Segatto *et al.* (2016)
	WUS intron	Segatto *et al.* (2016)

* Best substitution model estimated in jModelTest according to Akaike information criterion
[#] Combined sequences
