# Supplementary material for: Shades of white: The Petunia long corolla tube clade evolutionary history
Source: Genet Mol Biol. 2024 Feb 12;47(1):e20230279. doi: 10.1590/1415-4757-GMB-2023-0279 (PMC10882218; doi:10.1590/1415-4757-GMB-2023-0279)
Supplement: Table S3 - [file 1415-4757-GMB-47-01-e20230279-s3.pdf]

Genetics and Molecular Biology
https://doi.org/10.1590/1678-4685-GMB-2023-0279

Supplementary Material to "Shades of white: the Petunia long corolla tube clade evolutionary history"

Table S3 - GenBank accession number for DNA sequences used to obtain the phylogenetic tree for *Petunia* long corolla tube clade.

Lineages	trnH-psbA	trnS-trnG	rps12-rpl20	rpl32-trnL	matK gene	ITS	G3pdh gene	PolA1 gene	PID3C4	Hf1 gene	WUS intron	WOX4 intron
P. axillaris (A1)	JF917559	JF918116	**OR602738**	**OR602756**	AB262053	KJ200348	KJ507368	AB369404	KJ507305	AB244222	KF928403	KF928376
P. axillaris (A2)	JF917414	JF917970	**OR602739**	**OR602757**	**OR602746**	**OR602675**	**OR602770**	**OR602766**	**OR602751**	**OR602776**	**OR602781**	**OR602786**
P. parodii	JF917806	JF918364	**OR602742**	**OR602761**	AB262054	KJ200350	KJ507369	AB369408	KJ507306	AB244228	KF928420	KF928377
P. subandina	JF917863	JF918421	**OR602745**	**OR602764**	AB262055	KJ200351	KJ507370	AB369406	KJ507307	AB244221	KF928412	KF928385
P. exserta (E1)	**OR602731**	**OR602735**	-	**OR602759**	**OR602748**	**OR602679**	**OR602772**	**OR602765**	**OR602753**	**OR602778**	**OR602783**	**OR602788**
P. exserta (E2)	**OR602730**	**OR602733**	**OR602740**	**OR602758**	**OR602747**	**OR602678**	**OR602771**	**OR602767**	**OR602752**	**OR602777**	**OR602782**	**OR602787**
P. secreta	AY772897	KC832915	**OR602744**	**OR602763**	KJ507344	AY772937	KJ507383	KJ507364	KJ507323	KJ507348	KF928400	KF928373
P. sp1	**OR602733**	**OR602737**	**OR602743**	**OR602762**	**OR602750**	**OR602677**	**OR602774**	**OR602769**	**OR602755**	**OR602780**	**OR602785**	**OR602790**
P. sp3	**OR602732**	**OR602736**	**OR602741**	**OR602760**	**OR602749**	**OR602676**	**OR602773**	**OR602768**	**OR602754**	**OR602779**	**OR602784**	**OR602789**

Lineages	trnH-psbA	trnS-trnG	rps12-rpl20	rpl32-trnL	matK gene	ITS	G3pdh gene	PolA1 gene	PID3C4	Hf1 gene	WUS intron	WOX4 intron
P. occidentalis	KJ024584	KJ024580	-	-	AB262066	KJ200355	KJ507379	KJ507360	KJ507318	AB244234	KF928405	KF928378
P. integrifolia	DQ208110	DQ207980	MK208371	MK208404	AB262061	DQ208047	KJ507376	AB369412	KJ507314	AB244226	KF928415	KF928388
C. caesia	JX178636	JN565823	MK208341	MK208374	MK210204	MK424876	**OR602775**	MK433519	-	MK492810	MK492756	MK492836
C. parviflora	JX178654	DQ208029	MK208366	MK208399	KJ507347	DQ208030	KJ507384	KJ507366	-	KJ507349	KF928409	**OR602791**

- Missing data; codes in bold – sequences obtained in the current work
