# Supplementary material for: Shades of white: The Petunia long corolla tube clade evolutionary history
Source: Genet Mol Biol. 2024 Feb 12;47(1):e20230279. doi: 10.1590/1415-4757-GMB-2023-0279 (PMC10882218; doi:10.1590/1415-4757-GMB-2023-0279)
Supplement: Table S4 - [file 1415-4757-GMB-47-01-e20230279-s4.pdf]

*Genetics and Molecular Biology*
https://doi.org/10.1590/1678-4685-GMB-2023-0279

# Supplementary Material to "Shades of white: the Petunia long corolla tube clade evolutionary history"

**Table S4 -** Nuclear microsatellite markers used for genotyping *Petunia* long corolla tube clade.

| Chr | Locus | *Forward* | *Reverse* |
|---|---|---|---|
| 1 | PM188 | CCCAACCATTGGCTACAGCC | GGACAACACAATACAATCTCTGC |
| 1 | PM195 | GCCTTTCGCCGCTGTCACTG | GAGCAAATCGTGACCGTTGG |
| 2 | PM21 | CTACCGGTAGGCAGTAGTTGC | CCTCGACCTTCTTCCTGAC |
| 4 | PM8 | TCTGCAAACTTCAAAGCCAA | ACATGCCATGCACTTTTGAG |
| 4 | PM173 | CAGCGCTATCAACAGCAG | GTGAGAGGCAAGTGATTGG |
| 5 | PM167 | CTCACTAACCAACTTCACC | CTAAGAAGCTTAAGAGTG |
| 5 | PM177 | CCCTTACTCTCTTCTTCACC | GAACTATGAACCATAGCTCTC |

Chr – *Petunia hybrida* chromosome. All forward primers contained the M-13 tail **CACGACGTTGTAAAACGAC** sequence. Primers were described by Bossolini *et al.* (2011).
