# Supplementary material for: Shades of white: The Petunia long corolla tube clade evolutionary history
Source: Genet Mol Biol. 2024 Feb 12;47(1):e20230279. doi: 10.1590/1415-4757-GMB-2023-0279 (PMC10882218; doi:10.1590/1415-4757-GMB-2023-0279)
Supplement: Table S5 - [file 1415-4757-GMB-47-01-e20230279-s5.pdf]

Genetics and Molecular Biology
https://doi.org/10.1590/1678-4685-GMB-2023-0279

Supplementary Material to "Shades of white: the Petunia long corolla tube clade evolutionary history"

Table S5 - Number of individuals per lineage per DAPC group (best K = 3)

**Lineages**	**Group 1**	**Group 2**	**Group 3**
P. axillaris A1	1	0	9
P. axillaris A2	28	1	34
P. exserta E1	0	11	2
P. exserta E2	3	71	6
P. secreta	3	1	46
P. parodii	20	0	19
P. subandina	11	0	12
P. sp1	23	0	0
P. sp3	0	7	4
